# Genomic studies of envelope gene sequences from mosquito and human samples from Bangkok, Thailand

**DOI:** 10.1186/s40064-016-3634-y

**Published:** 2016-11-11

**Authors:** Pannamthip Pitaksajjakul, Surachet Benjathummarak, Hyun Ngoc Son, Supatra Thongrungkiat, Pongrama Ramasoota

**Affiliations:** 1 Center of Excellence for Antibody Research, Faculty of Tropical Medicine, Mahidol University, 420/6 Ratchathewi, Bangkok, 10400 Thailand; 2 Department of Social and Environmental Medicine, Faculty of Tropical Medicine, Mahidol University, Bangkok, Thailand; 3 Department of Medical Entomology, Faculty of Tropical Medicine, Mahidol University, Bangkok, Thailand

**Keywords:** Dengue virus serotype 2, Quasispecies, Human, Mosquitoes, Bangkok, Thailand

## Abstract

Dengue virus (DENV) is an RNA virus showing a high degree of genetic variation as a consequence of its proofreading inability. This variation plays an important role in virus evolution and pathogenesis. Although levels of within-host genetic variation are similar following equilibrium, variation among different hosts is frequently different. To identify dengue quasispecies present among two hosts, we collected patient samples from six acute DENV cases and two pools of *Aedes aegypti* mosquitoes and analyzed the genetic variation of regions of the viral envelope gene. Among human and mosquito samples, we found three major clusters originating from two subpopulations. Although several shared lineages were observed in the two hosts, only one lineage showing evidence of neutral selection was observed among two hosts. Taken together, our data provide evidence for the existence of a DENV quasispecies, with less genetic variation observed in mosquitoes than humans and with circulating lineages found in both host types.

## Background

Dengue virus (DENV) is a single-stranded positive-sense RNA virus belonging to genus *Flavivirus* of family *Flaviviridae* (De la Guardia and Lleonart 2014). This virus is the causative agent of dengue fever (DF), dengue hemorrhagic fever (DHF), and dengue shock syndrome (DSS), which is transmitted by infected female *Aedes aegypti* mosquitoes and seasonally epidemic in Thailand. In 2012, for example, total numbers of DF, DHF, and DSS cases were 39,392 (61.5 cases per 100,000 people), 37,798 (59/100,000), and 1321 (2.1/100,000), respectively (Corbel et al. 2013). DENV comprises a set of four closely related but genetically distinct serotypes (DENV1–4). These four serotypes show 25–40% variation based on amino acid sequences (Thai et al. 2012). Additional variation is present within each serotype, namely, 6–8% at nucleotide and 3% at amino acid levels, thereby giving rise to a diverse set of genotype lineages (Thai et al. 2012). Because of the nature of RNA viruses, DENV is composed of populations of closely related sequences, known as quasispecies, that display genetic variation relative to their master sequences (Kurosu et al. 2014).

The DENV genome is a single 11-kb RNA strand coding for capsid, membrane, and envelope (E) proteins and seven non-structural proteins (NS1, NS2A, NS2B, NS3, NS4A, NS4B, and NS5) (Qi et al. 2008). Of these 10 proteins, the E protein with its higher sequence heterogeneity is most frequently used to study DENV variation. The E protein consists of three functionally different structural domains (EDI–III). Because it is mainly involved in cell receptor binding, the EDIII domain is the focus of the present study. As the primary target under immune selection pressure, EDIII is the most heterogeneous region and is influenced by positive selection (Chao et al. 2005). Consequently, the variation in the EDIII region has been exploited for the characterization of dengue virus evolution in both human hosts and mosquito vectors (Thai et al. 2012; Kurosu et al. 2014; Lin et al. 2004; Wang et al. 2002).

Several previous studies have revealed that DENV isolated from different infection phases, patients, or hosts shows different levels of variation. For example, the mean diversity of DENV3 existing in human hosts and mosquitoes was found to be 0.38% (ranging from 0.15 to 0.59%) and 0.21%, respectively (Lin et al. 2004). In a study of DENV1-infected patient plasmas, however, intra-host variation as low as 0.0072 was observed (Thai et al. 2012). In 2014, DENV2 isolated from samples collected from acute patients from several provinces of Thailand showed some variations, with an average diversity of 0.145 in primary infections and 0.020 in secondary infections (Kurosu et al. 2014). Compared with the amount of information collected from human hosts, especially those isolates circulating in Thai DENV patients, knowledge of genetic variation in DENV2 obtained from *A. aegypti* mosquitoes, the primary transmission vector, is still limited.

In this study, we used clonal sequencing to identify the sequence variation of DENV2 isolated from *Aedes* mosquitoes comparing to that found in DENV2 isolated in 2010 from six dengue patients at the Hospital for Tropical Diseases, Bangkok, Thailand. The results of this comparative study of genetic variation between humans and mosquitoes may have implications for DENV evolution, overall fitness during viral transmission, and pathogenesis.

## Methods

### Preparation of human and mosquito dengue samples

Plasma samples from six acute dengue patients (i.e. 3–7 days after onset of fever) were collected from the Hospital for Tropical Medicine, Faculty of Tropical Medicine, Mahidol University, Bangkok, Thailand, in 2010. This study was approved by the ethical committee of the Faculty of Tropical Medicine, Mahidol University (MUTM 2011-017-01). The samples were confirmed to be positive for NS1 antigen and were identified as secondary infections by anti-dengue IgM and IgG antibodies using an immunochromatography kit (SD Bioline, Kyonggido, Korea) (Setthapramote et al. 2012).

Four samples of naturally infected mosquitoes from an area of Bangkok experiencing a dengue virus infection outbreak were previously collected in 2007–2008 and stored at −80 °C (Thongrungkiat et al. 2011). Thirty to forty mosquitoes were collected in each pooled sample and used for viral RNA preparation. To minimize sequencing artifacts during amplification, viral RNA was directly isolated from human plasma and mosquito homogenate samples. Each pool of mosquitoes was ground with a sterile micropestle in 200 µl of cold phosphate-buffered saline (pH 7.4). The samples were then centrifuged at 17,800×*g* and 4 °C for 30 min. The supernatant was used for RNA isolation or stored at −80 °C for further use.

### RNA isolation and cDNA synthesis

Viral RNA was isolated from 140 µl of collected human plasma and mosquito homogenates using a Viral RNA mini kit (Qiagen, Hilden, Germany) according to the manufacturer’s instructions. The isolated RNA was then used for cDNA synthesis as previously described (Yenchitsomanus et al. 1996) using dengue virus-specific primer DEUR (5′-GCTGTGTCACCCAGAATGGCCAT-3′) and a Superscript III cDNA synthesis system (Invitrogen, Carlsbad, CA, USA).

### Dengue virus detection and serotyping

Using E gene-specific cDNA obtained from the previous step as a template, dengue virus serotyping was performed with serotype-specific primers based on the E gene as previously described (Yenchitsomanus et al. 1996). Different domain-III sites of DENV1–4 were amplified using primers D1L/D1R, D2L/D2R, D3L/D3R, and D4L/D4R (Yenchitsomanus et al. 1996). For DENV2, the I270 to P384 region was amplified. Hi-fidelity DNA polymerase (Expand Hifidelity PCR System, Roche Diagnostics, Branchburg, NJ, USA) was used to minimize the incorporation of mismatched nucleotides. PCR amplification of 300 ng of E gene-specific cDNA was performed in a Bio-Rad C1000 thermal cycler under the following cycling conditions: denaturation at 95 °C for 3 min, followed by 35 cycles of denaturation at 95 °C for 1 min, annealing at 53 °C for 1 min, and extension at 72 °C for 1 min, with an additional final extension of 72 °C for 7 min. The PCR products were electrophoresed and visualized on a 1.2% agarose gel. Six human plasma and two mosquito samples that yielded a specific DENV2 band were further gel-purified with a Purelink Quick Gel Extraction kit (Invitrogen, USA.) and used for gene cloning.

### Cloning of the E gene

E-gene 346-bp fragments from DENV2-positive samples were cloned into a pGEM-T Easy TA cloning vector (Promega, Madison, WI, USA) and chemically transformed into DH5α *Escherichia coli*. White transformed colonies were randomly selected from LB/Amp/IPTG/X-gal agar plates and subjected to colony PCR to check for the presence of an insert. Using a Purelink Plasmid miniprep kit (Invitrogen), plasmid DNA was then isolated from overnight cultures of single positive clones grown in Luria–Bertani broth containing 100 µg ml^−1^ ampicillin. Six to 10 clones from each human sample and 14 clones from each mosquito sample were sequenced using M13 universal primers. The DENV2 E gene sequences generated from both humans and mosquitoes were submitted to the DNA Data Bank of Japan (DDBJ) under accession numbers LC030023–LC030105.

### Sequence analysis

The E-gene sequences of DENV2 isolated from human and mosquito samples were aligned and analyzed both at nucleotide and amino acid levels using Bioedit and ClustalW (Hall 1999). Mean diversity of each sample was calculated as the number of substitutions divided by the total number of analyzed sequences. All sequences were further aligned using ClustalW in MEGA 6.0 (Tamura et al. 2013). A phylogenetic analysis of the analyzed sequences together with reference strains of DENV2 genotypes (Asian I, Asian II, Asian-American, Cosmopolitan, and American) was performed with the maximum-likelihood (ML) method based on the Kimura 2-parameter model. Bootstrap probabilities were estimated with 1000 replications. The DENV2 E-gene sequences obtained from mosquito and human samples were compared. The within-group mean distance of each human and mosquito sample was also calculated using MEGA. Synonymous (dS) and non-synonymous (dN) substitution rates of each human and mosquito variant were calculated to assess positive selection with the codon-based Z-test of selection using the Nei-Gojobori method as implemented in MEGA 6.0.

## Results

### Sample preparation and clonal sequencing

Six human plasma samples, along with two mosquito pools that were positive for DENV2 according to nested PCR, were subjected to clonal sequencing. A total of 55 sequences from human-derived samples (10, 10, 9, 6, 10, and 10 clones from samples ID36, ID37, ID41, ID45, ID47, and ID50, respectively) and 28 sequences from mosquito-derived samples (14 clones each from mos56 and mos275) were analyzed.

### Analysis of sequence variation

**Fig. 1:**
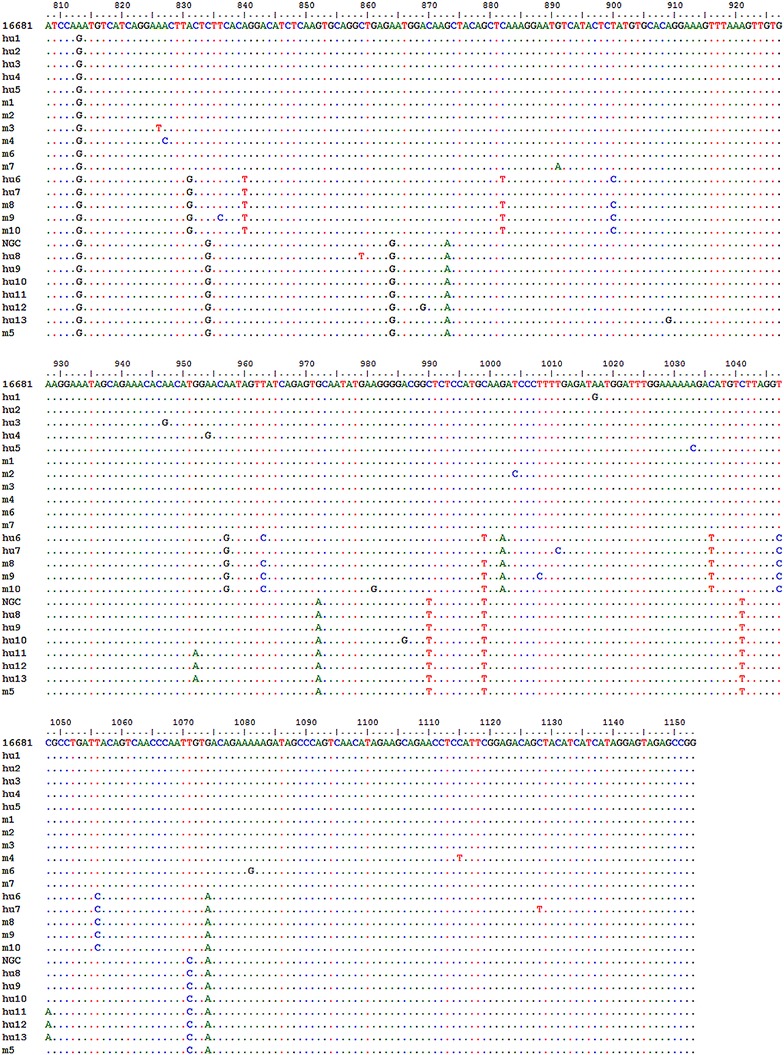
Nucleotide variation in human- and mosquito-derived sequences of the DENV2 envelope protein gene region. Nucleotides that differ from the corresponding nucleotide present in major population sequences hu2 and m1 are shown. The 13 variants hu1–hu13 are from human-derived samples, while m1–m9 correspond to nine variants from mosquito-derived samples. All the sequences were aligned with reference strains Thailand/16681/84, named as 16681, and New Guinea C (NGC), named as NGC

Nucleotide variation in the 346-bp E-gene region was analyzed with ClustalW and Bioedit programs (Fig. 1; Tables 1, 2). As shown in Fig. 1; Tables 1, 2, 55 clones from the six human plasma samples were classified as 13 different nucleotide variants (designated as hu1–hu13) on the basis of 382 nucleotide substitutions. Among these substitutions, 340 were silent and 42 were non-silent.

**Table 1:** Nucleotide and amino acid sequence diversity of the E-gene region of DENV-2 from infected patients and mosquitoes

Sample	Disease	No. of clones					Amino acid sequences		
			No. of substitutions^a^		Mean diversity (%)^c^	Mean distance (10^−2^)^c^	No. of substitutions^a^	Mean diversity (%)^b^	Mean distance (10^−2^)^c^
			Non-silent	Silent					
ID36	DF	10	2	0	2/3460 (0.058)	0.12	2	2/1150 (0.17)	0.35
ID37	DHF	10	10	110	120/3460 (3.47)	0.00	10	10/1150 (0.87)	0
ID41	DHF	9	9	81	90/3114 (2.89)	0.00	9	9/1035 (0.87)	0
ID45	DF	6	1	55	56/2076 (2.69)	0.19	1	1/690 (0.14)	0.29
ID47	DHF	10	0	2	2/3460 (0.058)	0.12	0	0/1150 (0.00)	0
ID50	DHF	10	21	91	112/3460 (3.24)	0.12	21	21/1150 (1.83)	0.17
Humans		55	42	340	382/19030 (2.01)		42	42/6325 (0.66)	
Mos56		14	12	112	124/4844 (2.56)	1.69	12	12/1610 (0.75)	0.63
Mos275		14	5	18	23/4844 (0.47)	0.89	5	5/1610 (0.31)	0.61
Mosquitoes		28	17	130	147/9660 (1.52)		17	17/3220 (0.53)	

*DF* dengue fever, *DHF* dengue hemorrhagic fever

^a^Number of substitutions relative to master sequences hu2 and m1

^b^Number of nucleotide (or amino acid) substitutions divided by the total number of sequenced nucleotides (or amino acids) multiplied by 100

^c^Calculated by pairwise comparison of nucleotide or amino acid sequences between clones within each sample using within- and between-group mean distances in MEGA

**Table 2:** Nucleotide sequence variants of the 346-bp E-gene region isolated from human plasma and mosquito samples compared with their corresponding master sequences (hu2 and mo1)

Sequence variant	No. of clones (% of total)	Clones	No. of nucleotide substitutions^a^	Total no. of substitutions^b^	dN/dS^c,d^
hu1	1 (1.80)	**36-**2	1	1	0.16
hu2	16 (29.1)	**36**-3, 4, 6, 8, 9, 10, 11, 12 **47**-2, 3, 5, 6, 7, 8, 9, 10	0	0	1.00
hu3	1 (1.8)	**36**-14	1	1	0.16
hu4	1 (1.8)	**47**-4	1	1	1.00
hu5	1 (1.8)	**47**-1	1	1	1.00
hu6	10 (18)	**37**-1, 2, 3, 4, 5, 6, 8, 10, 16, 18	12	120	
hu7	9 (16.4)	**41**-2, 4, 9, 18, 23, 24, 26, 27, 28	10	90	1.00
hu8	1 (1.8)	**45-**1	10	10	1.00
hu9	4 (7.3)	**45**-5, 6, 14, 25	9	36	1.00
hu10	1 (1.8)	**45-**15	10	10	1.00
hu11	8 (14.5)	**50**-2, 3, 4, 6, 7, 8, 9, 10	11	88	1.00
hu12	1 (1.8)	**50**-1	12	12	1.00
hu13	1 (1.8)	**50-**5	12	12	1.00
Total	55			382	
m1	11 (39.3)	**56**-4, 7, 15 **275**-24, 25, 27, 30, 32, 33, 35, 36	0	0	1.00
m2	1 (3.5)	**56**-20	1	1	0.16
m3	1 (3.5)	**275**-26	1	1	0.16
m4	1 (3.5)	**275**-31	2	2	0.08
m5	2 (7.1)	**275**-37, 38	9	18	1.00
m6	1 (3.5)	**275**-39	1	1	0.16
m7	1 (3.5)	**275**-40	1	1	0.16
m8	8 (28.6)	**56**-2, 5, 6, 10, 12, 13, 14, 17	12	96	1.00
m9	1 (7.1)	**56**-3	14	14	1.00
m10	1 (3.5)	**56**-8	13	13	1.00
Total	28			147	

^a^Number of substitutions relative to master sequences hu2 and mo1

^b^Total number of substitutions in each variant multiplied by the number of clones of that variant

^c^Ratio of non-synonymous to synonymous substitutions per site, calculated using the codon-based test of positive selection in MEGA

^d^ *p* value = 0.05

In mosquitoes, 10 different nucleotide variants (m1–m10) were identified (Fig. 1; Table 2). Among 28 analyzed sequences, we found 147 nucleotide substitutions (124 from mos56 and 23 from mos275). Of these, 130 substitutions were silent (112 from mos56 and 18 from mos275) and 17 were non-silent (12 from mos56 and 5 from mos275) (Table 1).

As shown in Table 1, the genetic diversity of each sample, which was based on mean diversity compared with the master nucleotide sequence, ranged from 0.058 to 3.47 for humans and 0.47 to 2.56 for mosquitoes. Within-group mean distance of each sample, calculated to study the genetic variation within individual samples, varied from 0.00 to 0.19 and 0.89 to 1.69 for human and mosquito samples, respectively.

**Fig. 2:**
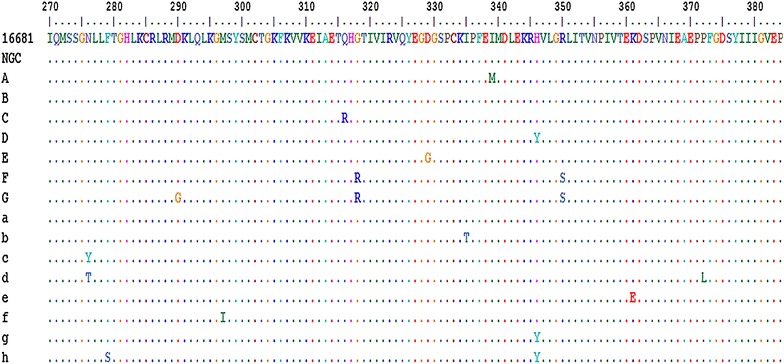
Amino acid variation in 55 human- and 28 mosquito-derived DENV2 envelope protein sequences. A and B represent seven variants from human-derived samples, while a–h correspond to eight variants from mosquito-derived samples

We also analyzed deduced amino acid sequences to determine the degree of sequence variation at the amino acid level (Fig. 2). Seven amino acid variants (A–G) were identified in the human samples, while eight variants (a–h) were observed in the mosquito samples (Table 3). Of 59 identified amino acid substitutions, 42 and 17 substitutions were derived from human and mosquito samples, respectively (Tables 1; 3). Overall mean diversities in human and mosquito samples were 0.66 and 0.53, respectively, with mean distances ranging from 0.00 to 0.35 for human-derived samples and 0.61 to 0.63 for mosquito-derived samples (Table 1). The Nei-Gojobori test of selection uncovered evidence for neutral selection in most of the variants; the exceptions were hu1, hu3, m2, m3, m4, m6, and m7, for which dN/dS was lower than 1 (Table 2).

**Table 3:** Amino acid sequence variants of the 115-amino-acid region of the E protein isolated from human plasma (A–G) and mosquito (a–h) samples compared with their corresponding master sequences (B and a)

Sequence variant	No. of clones (% of total)	Clones	No. of amino acid substitutions^a^	Type of amino acid substitution^b^
A	1 (1.8)	**36**-2	1	I339M
B	23 (42)	**36**-3, 4, 6, 8, 9, 10, 11, 12 **45**-1, 5, 6, 14, 25 **47**-1, 2, 3, 4, 5, 6, 7, 8, 9, 10	0	–
C	1 (1.8)	**36**-14	1	Q316R
D	19 (34.5)	**37**-1, 2, 3, 4, 5, 6, 8, 10, 16, 18 **41**-2, 4, 9, 18, 23, 24, 26, 27, 28	1	H346Y
E	1 (1.8)	**45**-15	1	D329G
F	9 (16.4)	**50**-2, 3, 4, 5, 6, 7, 8, 9, 10	2	G318R, R350S
G	1 (1.8)	**50**-1	3	D290G, G318R, R350S
Total	55		9	
a	13	**56**-4, 7, 15 **275**-24, 25, 27, 30, 32, 33, 35, 36, 37, 38	0	–
b	1	**56**-20	1	I335T
c	1	**275**-26	1	N276Y
d	1	**275**-31	2	N276T, P372L
e	1	**275**-39	1	K361E
f	1	**275**-40	1	M297I
g	9	**56**-2, 5, 6, 8, 10, 12, 13, 14, 17	1	H346Y
h	1	**56**-3	2	F279S, H346Y
Total	28		9	

^a^Number of substitutions relative to master sequences B and a

^b^I, isoleucine; T, threonine; N, asparagine; Y, tyrosine; P, proline; L, leucine; K, lysine; E, glutamic acid; M, methionine; H, histidine; F, phenylalanine, S, serine; Q, glutamic acid; R, arginine; D, aspartic acid; G, glycine

### Phylogenetic analysis

**Fig. 3:**
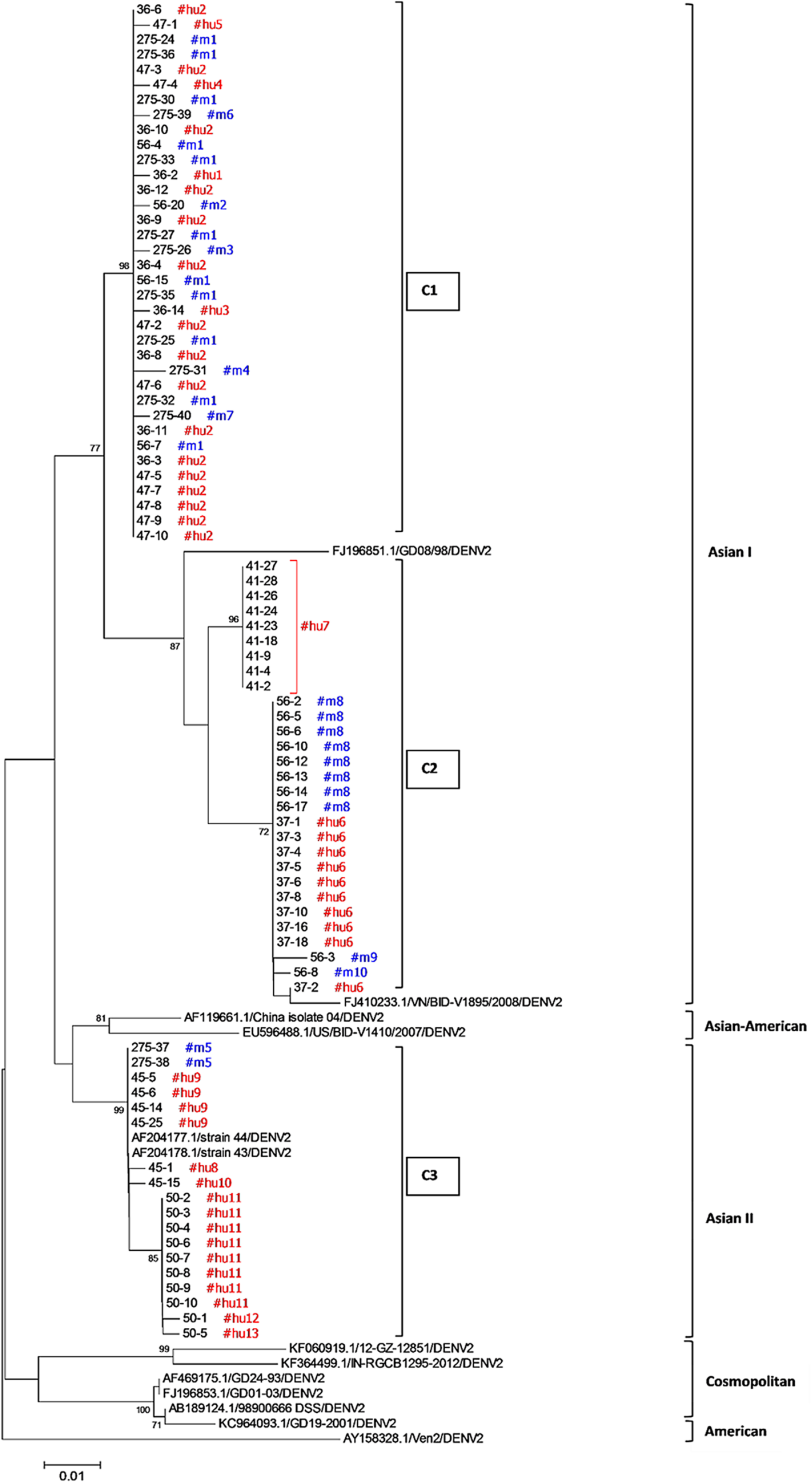
Neighbor-joining phylogenetic tree of the 346-bp region of the dengue virus envelope gene from 28 mosquito- and 55 human-derived sequences. Bootstrap support percentages based on 1000 replicates are indicated at nodes

Phylogenetic analysis separated the DENV2 nucleotide sequences from mosquito- and human-derived samples into three clusters (C1, C2, and C3) (Fig. 3). Cluster C1 constituted the largest population, having five human nucleotide variants (hu1, hu2, hu3, hu4, and hu5) derived from two human samples (ID36 and ID47) as well as six mosquito nucleotide variants (m1, m2, m3, m4, m6, and m7). Variant m1, the major sequence from mosquitoes (Table 2), was identical to 16 of 55 clones of human variant hu2. Cluster C2 contained three nucleotide variants from mosquito (m8, m9, and m10) derived mainly from mos56 and two human variants (hu6 and hu7) derived from human samples ID37, and ID41. Variant m8, the second most abundant sequence, was identical to 10 clones of variant hu6. Although representing distinct DENV2 lineages, clusters C1 and C2 were both most closely related to the Asian I genotype (FJ196851.1 and FJ10233.1), and classified in the same suppopulation. The third cluster, C3, comprised two clones of mos275 (275-37 and 275-38) classified as variant m5 and clones from human samples ID45 and ID50 identified in six variants (hu8, hu9, hu10, hu11, hu12, and hu13). Members of C3 were closely related to DENV2 Asian II genotype (AF204177.1 and AF204178.1).

## Discussion and conclusions

Flaviviruses, including DENV, are RNA viruses known to exist within hosts as a mixture of genetically related variants. When undergoing replication, these viruses may generate new progeny having nucleotide and amino acid mutations relative to the master sequence. A correlation is hypothesized to exist between these variations and viral pathogenesis as well as viral evolution (fitness) within and between hosts (Lin et al. 2004; Kurosu 2011; Jerzak et al. 2005). In this study, we therefore compared both inter- and intra-host genetic diversity of the EDII–III region of DENV2 from mosquito and human samples.

Six human plasma samples and two (mos56 and mos275) of four collected mosquito pools displayed 346-bp PCR products specific to the DENV2 E gene target region. The sequence populations represented in these human and mosquito samples were then identified by clonal sequencing. In contrast to a previous study of human plasma samples (Wang et al. 2002), we found a higher number of silent than non-silent mutations in the 83 sequences analyzed. None of the clones had a stop codon in this region.

Comparison of DENV2 sequences obtained from humans and mosquitoes revealed a slightly lower diversity (inferred from the overall mean diversity) in the mosquito samples (Table 1), a finding similar to observations of previous studies (Chao et al. 2005; Lin et al. 2004). From the spectrum of viral sequence variation found in mosquitoes, this genetic diversity was apparent even during a short incubation period of 8–12 days (Lin et al. 2004). In the future, determination of genetic variation within individual mosquitoes might be required, but the occurrence of genetic mutations during viral amplification of single mosquitoes during cell culture experiments will need to be considered (Craig et al., 2003).

As reported previously, DENV populations during secondary infection are more homogeneous (0.010–0.037% variation) than viruses isolated from primary infections (0.067–0.222%) (Kurosu et al., 2014). In the present study, human samples were all derived from secondary DENV infections (Setthapramote et al. 2012). Nevertheless, the mean diversity of DENV2 nucleotide sequences isolated from our human samples ranged from 0.058 to 3.47%, indicating higher variation between different samples than that previously reported (Kurosu et al. 2014; Lin et al. 2004; Wang et al. 2002). This higher variation, however, was inferred from mean diversity compared with master sequences. When individual human samples were considered, we found that the different human samples were infected with DENV2 strains belonging to different clades. Within-group mean distance was then calculated. More homogeneous populations were subsequently identified within each individual human sample (0.00–0.19) (Table 1). In regard to mosquito-derived samples, heterogeneous populations were identified (mean distance 0.89 for Mos275 and 1.69 for Mos56) from the individual samples resulting from a collection of mosquitoes in each pooled sample.

As most uncovered substitutions were silent, the variation in amino acid sequences was less heterogeneous. In particular, a mean diversity of 0.00–1.83 and 0.31–0.75 were calculated for human- and mosquito-derived samples, respectively.

Notably, amino acid substitutions observed in DENV from mosquito samples were different from those obtained from human samples (Table 3). This difference suggests that the amino acid substitutions occurred at random positions in both host types. Interestingly, substitution at H346Y, previously found in DENV2 from human plasma samples (Puiprom et al. 2010), was also observed in our study in both human- and mosquito-derived sequences. This occurrence suggests that the variation found at this position is not due to the natural escape of DENV2 from immune selection pressures occurring in the hosts; instead, it is probably derived from variation in DENV2 itself. This H/Y variant may thus have been transmitted to human hosts by infected mosquitoes.

Regarding the 13 and 10 nucleotide variants respectively cloned from human- and mosquito-obtained samples, the majority of sequences were identical to the consensus sequence. Hu2 variant which constituted the largest population and was mostly derived from two dengue patients (ID36 and ID47). Regarding mosquito-derived sequences, variant m1 was the master sequence among two mosquito samples (11 of 28: 8 from mos275 and 3 from mos56). Both of the largest populations, hu2 and m1, were classified in the same clade (C1) (Fig. 3). Several other minor variants were identified within this clade: hu1, hu3, hu4, hu5, m2, m3, m4, m6, and m7. Variants hu6 and hu7, derived from dengue patients ID37 and ID41, were the second and the third most abundant variants, respectively, while m8 was the second most abundant variant (8/28) isolated from mosquito samples. These variants from humans (hu6 and hu7) and mosquitoes (m8) were grouped in clade C2 together with minor variants m9 and m10 (Fig. 3). Among 14 sequences obtained from mos56, two major lineages, classified into clades C1 and C2, were responsible for the greater variation and higher mean diversity of mos56 (Tables 1, 2). The third clade (C3) contained only one variant from mosquitoes (m5) along with six human variants (hu8, hu9, hu10, hu11, hu12, and hu13) derived from the other two dengue patients (ID45 and ID50). Among six dengue patients, three major clusters (C1, C2, and C3), each representing samples from two dengue patients, were clearly identified. Of the six individual dengue patient samples, four (ID36, 45, 47, and 50) contained three variants (Table 2) and two (ID37 and ID41) were represented by identical sequence among all clones analyzed. Consequently, dengue virus quasispecies were present in all individual human samples except for ID37 and ID41. To further confirm the existence of dengue quasispecies in human samples, however, a higher number of clonal sequences need to be isolated from each sample.

To test for genetic fitness on the basis of positive selection, we used MEGA to determined dN/dS of each human and mosquito variant. Selection for variants m1 and hu2 was shown to be neutral (dN/dS = 1.00), suggesting that these two variants are the optimal DENV2 sequences circulating among the two hosts. Some other variants (hu1, hu3, hu4, hu5, m1, m2, m3, m4, m6, and m7) derived from these two variants were found to have minimal fitness, a conclusion inferred partly from the deleterious effect implied by a dN/dS lower than 1 (Table 2). We found that all variants in the C2 cluster were experiencing neutral selection (dN/dS = 1) (Table 2; Fig. 3). Similarly, members of the C3 cluster, namely, hu8, hu9, hu10, hu11, hu12, and hu13, also showed evidence of neutral selection (Table 2).

Even though the E-gene region is frequently used to study quasispecies, other structural and nonstructural genes, such as those encoding NS1, NS3, NS5 or the capsid, should not be excluded from consideration in future studies (Chao et al. 2005; Lin et al. 2004). To our knowledge, however, this is the first reported comparison of the genetic variation of DENV2 quasispecies circulating in humans and mosquitoes in Bangkok, Thailand. To verify the presence of quasispecies within these two hosts, a larger sampling of both humans and mosquitoes will be required in future studies.
